# Evaluation of a Novel Magneto-Optical Method for the Detection of Malaria Parasites

**DOI:** 10.1371/journal.pone.0096981

**Published:** 2014-05-13

**Authors:** Ágnes Orbán, Ádám Butykai, András Molnár, Zsófia Pröhle, Gergö Fülöp, Tivadar Zelles, Wasan Forsyth, Danika Hill, Ivo Müller, Louis Schofield, Maria Rebelo, Thomas Hänscheid, Stephan Karl, István Kézsmárki

**Affiliations:** 1 Department of Physics, Budapest University of Technology and Economics, Budapest, Hungary; 2 Department of Oral Biology, Semmelweis University, Budapest, Hungary; 3 Infection and Immunity Division, Walter and Eliza Hall Institute of Medical Research, Parkville, Victoria, Australia; 4 Department of Medical Biology, University of Melbourne, Parkville, Victoria, Australia; 5 Queensland Tropical Health Alliance Australian Institute of Tropical Health and Medicine James Cook University, Douglas, Queensland, Australia; 6 Instituto de Medicina Molecular, Faculdade de Medicina, Lisbon, Portugal; 7 Condensed Matter Research Group of the Hungarian Academy of Sciences, Budapest, Hungary; University of Melbourne, Australia

## Abstract

Improving the efficiency of malaria diagnosis is one of the main goals of current malaria research. We have recently developed a magneto-optical (MO) method which allows high-sensitivity detection of malaria pigment (hemozoin crystals) in blood via the magnetically induced rotational motion of the hemozoin crystals. Here, we evaluate this MO technique for the detection of *Plasmodium falciparum* in infected erythrocytes using in-vitro parasite cultures covering the entire intraerythrocytic life cycle. Our novel method detected parasite densities as low as ∼40 parasites per microliter of blood (0.0008% parasitemia) at the ring stage and less than 10 parasites/*µ*L (0.0002% parasitemia) in the case of the later stages. These limits of detection, corresponding to approximately 20 pg/*µ*L of hemozoin produced by the parasites, exceed that of rapid diagnostic tests and compete with the threshold achievable by light microscopic observation of blood smears. The MO diagnosis requires no special training of the operator or specific reagents for parasite detection, except for an inexpensive lysis solution to release intracellular hemozoin. The devices can be designed to a portable format for clinical and in-field tests. Besides testing its diagnostic performance, we also applied the MO technique to investigate the change in hemozoin concentration during parasite maturation. Our preliminary data indicate that this method may offer an efficient tool to determine the amount of hemozoin produced by the different parasite stages in synchronized cultures. Hence, it could eventually be used for testing the susceptibility of parasites to antimalarial drugs.

## Introduction

Although research activities aim at developing novel methods for high-sensitivity diagnosis of malaria, only a few of these approaches are feasible for clinical and in-field diagnosis. The two main diagnostic methods currently in practice are the antigen-based detection of malaria parasites using rapid diagnostic tests (RDT) and the microscopic observation of infected red blood cells in blood smears [1]–[4]. The detection limits of RDT and light microscopy have been reported to be approximately 100 parasites/*µ*L and 5–50 parasites/*µ*L, respectively [1], [5]–[7], corresponding to parasitemia levels of around 0.002% and 0.0001–0.001%. RDT are becoming more affordable, however, they cannot provide a quantitative measure of parasitemia. Presently they do not possess sufficient sensitivity to detect low-level infections which are very common in endemic settings along with the false positive samples due to the presence of parasite protein (HRP2) even after resolving the infection. On the other hand, light microscopy is time and labor intensive and the detection threshold of 5 parasites/*µ*L is rather limited to ideal conditions such as good-quality blood films, highly trained microscopists and high-powered microscopes; most of which are rarely present in real life practice. Most routine diagnostic laboratories achieve approximately 50 parasites/*µ*L and can detect about 50% of malaria cases [6], [8], [9].

Molecular methods such as polymerase chain reaction (PCR) assays surpass the performance of RTD and light microscopy [10], [11]. However, they often require expensive equipment and reagents, highly trained laboratory personnel and are prone to contamination [12]. Although real-time PCR has a detection limit corresponding to a few parasites per *µ*L of blood [13], [14], it is not yet a practical method for routine diagnosis under field conditions.

The idea to take advantage of the unique magnetic properties of malaria pigment (hemozoin) and to use it as an alternative target of optical diagnosis has been proposed by several groups [15]–[19]. Hemozoin is a micro-crystalline heme compound produced by malaria parasites as they detoxify free heme derived from hemoglobin digestion [20]. Our recent study using synthetic hemozoin crystals suspended in blood demonstrated that the rotating-crystal magneto-optical (MO) diagnostic method can detect hemozoin concentrations down to 15 pg/*µ*L [15]. Using data describing the rate of hemoglobin digestion by *P. falciparum* available in the literature [21]–[23], this threshold concentration was estimated to be equivalent to a parasite density of ≤30 parasites/*µ*L in infected blood provided that the entire amount of hemozoin produced by the parasites is released into the lysed cell suspension.

However, the situation in malaria infection and thus the requirements for diagnostic applications are different. One aspect is that in *Plasmodium falciparum* infections only early developmental forms, such as rings and early trophozoites, are usually found in the peripheral circulation, since later developmental stages sequester in the capillaries [24]. Furthermore, the shape and size of hemozoin crystals may be different for synthetic and naturally grown crystals and also depend on the developmental stages of the parasites as well as the parasite species [25]–[27]. Since the MO method detects only hemozoin crystals released into suspension which can be magnetically rotated, the aggregation of the crystals or their binding to other components of lysed blood could influence the sensitivity of this technique.

In the present study we aimed to address these issues by evaluating the performance of the MO technique using synchronized cultures of *P. falciparum* and to investigate the limit of detection in samples with low levels of parasitemia, which would establish its potential usefulness for field trials. (For a short description of the MO approach see the Materials and Methods section.).

## Results and Discussion

### Parasite Cultures

**Figure 1:**
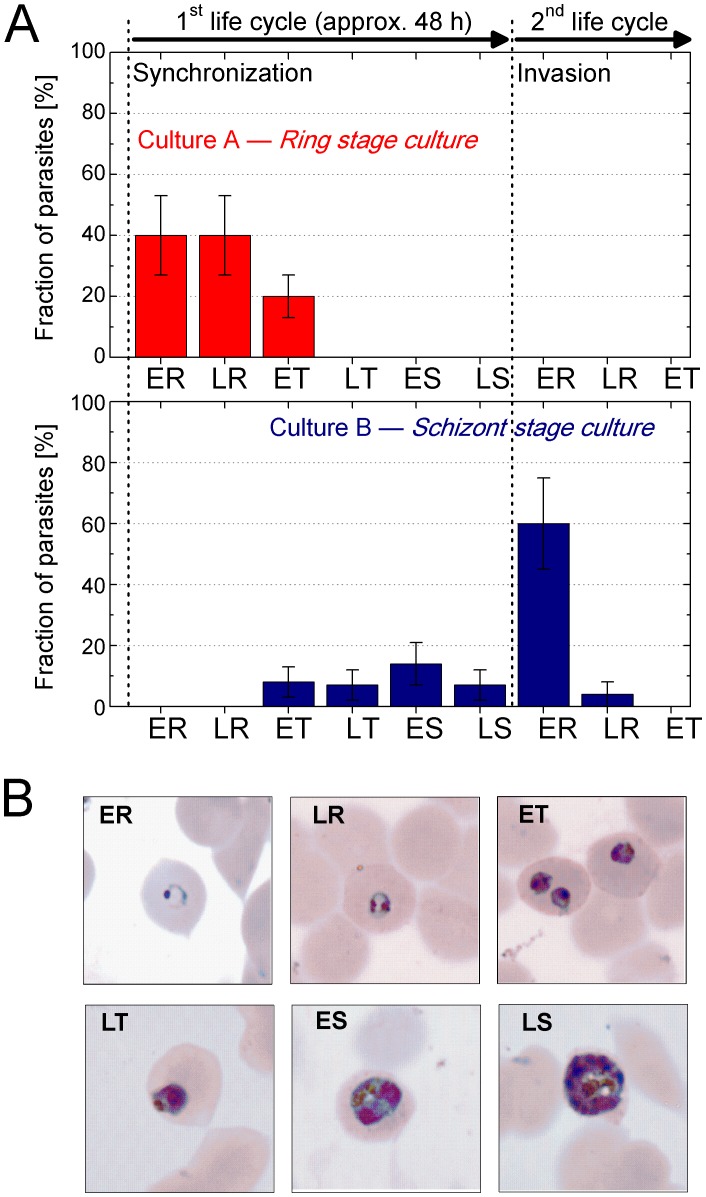
Distribution of parasite life cycle stages in the two *Plasmodium falciparum* cultures used in the present study. Panel A: The *ring stage culture* contained early rings, late rings and some early trophozoites of the first generation after synchronization. The *schizont stage culture* was on the verge of the first and second life cycles where most of the schizont stages have already turned to early ring stages of the second generation following invasion. Therefore, the ring stage culture contained only the hemozoin present in the parasites up to the early trophozoite stage, while the schizont stage culture had the entire hemozoin content formed during one generation of parasites with the largest portion produced by schizonts. Panel B: Light microscopy images of Giemsa stained thin blood films containing infected red blood cells with parasites in different stages of maturity (taken from these two cultures). In both panels the labels ER, LR, ET, LT, ES and LS correspond to early-ring, late-ring, early-trophozoite, late-trophozoite, early-schizont and late-schizont stages, respectively.

We investigated the sensitivity and detection threshold of the rotating-crystal MO method using two cultures (A and B) with different maturity distributions of the parasites. The distributions of the parasites among the different stages – early-ring, late-ring, early-trophozoite, late-trophozoite, early-schizont and late-schizont stages – in the two cultures are displayed in Fig. 1 together with light microscopy images of parasites representative of these stages.

Culture A had a total parasite density of P
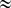
3.1×10^5^ parasites/*µ*L. Parasite stage distribution corresponded to the beginning of the parasite life cycle following synchronization with mostly ring stages and some early trophozoites. This culture, referred to as the *ring stage culture* in the following, is representative to the distribution of parasite blood stages most often encountered in natural *P. falciparum* infections.

Culture B had a lower total parasite density of P
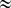
2.8×10^4^ parasites/*µ*L. Parasite stage distribution corresponded to the verge of the first and second parasite life cycles where some of the parasites were still in the schizont form but most of them, following invasion, already turned to early-stage rings of the next generation. Since the main hemozoin content present in this culture was formed during the first life cycle with a dominant contribution from schizont stage parasites [21]–[23], we will refer to it as the *schizont stage culture*. Schizont stage parasites are not normally found in the peripheral blood during human *P. falciparum* infection due to their sequestration in small capillary blood vessels [24]. However, this stage restriction is not present in other, non-sequestering, species of human malaria parasites such as *P. vivax* [28].

### Limit of Detection

**Figure 2:**
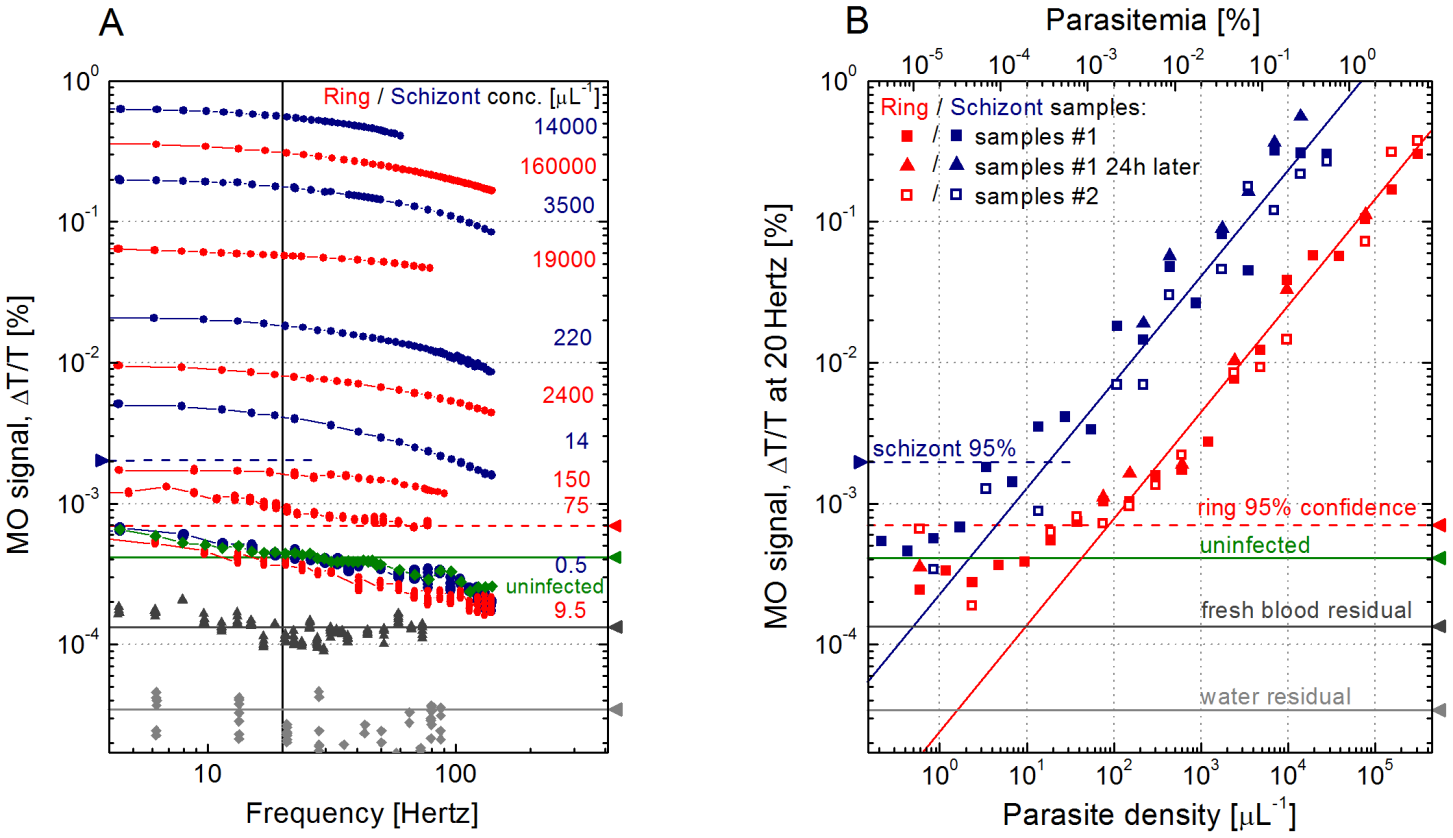
Magneto-optical (MO) detection of parasitemia in synchronized *Plasmodium falciparum* cultures. Panel A: Red and blue curves show the frequency dependent MO signal for samples from the ring and schizont stage cultures, respectively, with various levels of parasite density given in *µ*L^−1^ units on the right of the respective curves. The green curves shows the signal from uninfected reference samples. Data plotted with triangles and diamonds are the residual signal from freshly hemolyzed uninfected blood and water, respectively. The frequency scale corresponds to the rotation speed of the magnetic field. Panel B: Red and blue squares in panel B are the MO signal values measured at 20 Hz – indicated by a vertical solid line in panel A – for the dilution series prepared from the original ring and schizont stage cultures, respectively. Solid and open squares correspond to the duplicate samples labeled as samples #1 and samples #2. Triangles indicate the results obtained by remeasuring samples #1 with 24 h delay. The solid lines following the trend of the MO signal at higher parasite densities for ring (red line) and schizont (blue line) samples are guides for the eye. For ring and schizont stage samples with parasite densities lower than 10 parasites/*µ*L and 1 parasites/*µ*L, respectively, the MO signal does not further decrease. The green horizontal line shows the residual MO signal of uninfected blood, which is the mean detection limit of our method. The 95% confidence levels of this mean detection limit for the ring and schizont stage samples are indicated by red and blue dashed lines, respectively. Correspondingly, for ring and schizont stage samples with parasite density higher than 40 parasites/*µ*L and 10 parasites/*µ*L, respectively, the diagnosis is positive with a confidence of at least 95%. The background signal for freshly hemolyzed uninfected blood and water are also shown by dark and light grey lines. All these horizontal indicators are also shown in panel A for reference. The upper horizontal scale shows the corresponding levels of parasitemia.

The MO signal, the measure of hemozoin content within the lysed cell suspension, is shown in Fig. 2 for dilution series of culture A and culture B. The 20 serial 2-fold dilutions of the two cultures using uninfected erythrocytes allowed for the MO signal to be assessed over 6 orders of magnitude of parasitemia. As a general trend in Fig. 2A, the MO signal varies proportionally to the parasitemia level and the signal for each sample shows a gradual decrease with increasing frequencies of the rotating magnetic field. This frequency dependence is in agreement with previous results obtained for synthetic hemozoin crystals suspended in blood and originates from the viscosity of the lysed cell suspension hindering fast rotations of the crystals [15].

Samples from the dilution series of culture B exhibited higher MO signals as compared to dilutions of culture A with the same level of parasitemia reflecting the increase in intracellular hemozoin content over the course of the parasite life cycle. Although the overall frequency dependence of the MO signal is similar for the two cultures, the more pronounced decrease with increasing frequency found for the dilutions of culture B is likely due to the larger crystal size formed in schizont stage parasites.

In order to determine the limit of detection limit for our method, the MO signal at 20 Hz was plotted versus parasitemia (and parasite density) in Fig. 2B for the dilution series of the ring and schizont stage cultures. For reference, we also measured uninfected blood samples using the same protocol. These showed a residual MO signal of 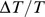 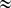
4×10^−4^ % in average as indicated in Fig. 2B. This value corresponds to the mean limit of detection of our method. At low levels of parasite density (*P*), namely for samples with P≤10 parasites/*µ*L from the dilution series of the ring stage culture, the signal does not further decrease with decreasing parasitemia but approaches the residual level observed for the uninfected samples. Hence, this residual MO signal is not related to hemozoin. We note that the frequency dependence of the residual MO signal differs from the hemozoin-dependent MO signal dominant at higher concentrations. (See curves corresponding to the ring stage sample with *P* = 9.5 parasites/*µ*L, the schizont stage sample with *P* = 0.5 parasites/*µ*L and the uninfected samples in Fig. 2A.).

For highly diluted ring stage samples with *P*≤10 parasites/*µ*L, where the MO signal is independent of the parasite density, we found that the standard deviation of the MO signal from the mean limit of detection (represented by the signal level of uninfected samples) is 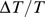 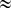
1.6×10^−4^ %. Assuming Gaussian distribution for the residual MO signal values, the 95% confidence level of the mean limit of detection for ring stage samples is 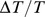 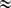
7.2×10^−4^ % corresponding to a parasite density of ∼40 parasites/*µ*L. This is equivalent to a parasitemia level of 0.0008%. The scattering of the data is larger for schizont stage samples. In that case we estimated the 95% confidence level of the mean limit of detection to be 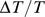 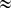
2×10^−3^ % corresponding to a parasite density of ∼10 parasites/*µ*L or a parasitemia of 0.0002%.

The presence of a frequency-dependent residual MO signal indicates that some components of lysed blood can be magnetically oriented and rotated similarly to the hemozoin crystals. We have also studied freshly drawn blood following the same lysis protocol. The residual MO signal observed in that case was considerably lower than found for either the ring or schizont stage samples, which were measured following the freeze-thaw-lysis procedure (see Fig. 2 and also Fig. S1 of the Supporting Information S1). The noise floor of our equipment, shown for pure water in Fig. 2, is nearly frequency independent and about one order of magnitude smaller than the residual signal from fresh blood. This enables further improvement of the detection limit provided that the residual MO signal from blood can be reduced by optimizing blood sample treatment.

Sequential MO measurements performed on the same sample at different time intervals up to one hour gave identical results. In several cases we confirmed the reproducibility of the protocol by repeating the measurement for duplicate samples labeled as samples #1 and samples #2 in Fig. 2B. When samples were re-measured after being stored for 24 hours at 4°C and sonicated for 30 minutes prior to the MO measurements, typically an increase of the MO signal of not more than 10–30% was observed. Additional data about the effect of sonication on the MO signal, given in Fig. S1 of the Supporting Information S1, show that sonication increases the MO signal. We found that the elimination of the entire freeze-thaw-sonication process from the protocol reduces the MO signal by 30–40%. In this case, the lowest level of parasitemia still detectable with a confidence of 95% is approximately 0.0012% for ring stage parasites. Thus, the use of the clearing solution alone appears to be sufficient if fresh blood samples are used. Nevertheless, it seems beneficial to use sonication, where available, especially when screening samples with very low parasitemias.

### Hemozoin Concentration of the Cultures

The hemozoin content of the two cultures can be roughly estimated from the parasite density and the stages of parasite development specified in Fig. 1. Ring and early trophozoite stages respectively convert about 3–5% and 15–20% of the total hemoglobin in the infected red blood cells to hemozoin [21], [22]. Parasites in the schizont stage convert about 50–70% of hemoglobin to hemozoin [20], [29], [30]. Using these hemoglobin conversion rates, we calculated the hemozoin concentrations of the undiluted ring and schizont stage cultures and compared them to the amounts estimated based on our MO reference data previously obtained for synthetic hemozoin [15]. These values are listed in Table 1. According to the estimate based on the MO signal, the lowest hemozoin concentration still measurable by our MO setup using the present protocol is ∼20 pg/*µ*L, which is close to the threshold reported for artificial crystals [15]. (This concentration refers to the original hemozoin content of blood samples before the 20-fold dilution performed prior to MO measurements.).

**Table 1:** Using the hemozoin conversion rates reported in the literature for the different parasite stages (rings: 3–5%, trophozoites: 15–20%, and schizonts: 50–70%) [20]–[22], [29], [30] and the stage distribution of the parasites (Fig. 1), we estimated the hemozoin content of culture A (ring stage culture) and culture B (schizont stage culture).

	Estimated hemozoin contentbased on literature	Estimated hemozoin contentfrom MO measurements
Culture A (P = 3.1×10^5^ parasites/*µ*L)	17–25 ng/*µ*L	6 ng/*µ*L
Culture B (P = 2.8×10^4^ parasites/*µ*L)	14–23 ng/*µ*L hemozoin	9 ng/*µ*L

The lower and upper values of the hemozoin content correspond to the lower and upper values of the conversion rates quoted above. Note that the cultures have different parasite densities. We also estimated the hemozoin concentration of the two cultures based on MO signal using the conversion factor c_HZ_ = 1 ng/*µ*L → 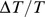 = 1.4% between the hemozoin concentration and the low-frequency (∼1 Hz) MO signal previously determined for artificial hemozoin crystals suspended in blood [15].

**Figure 3:**
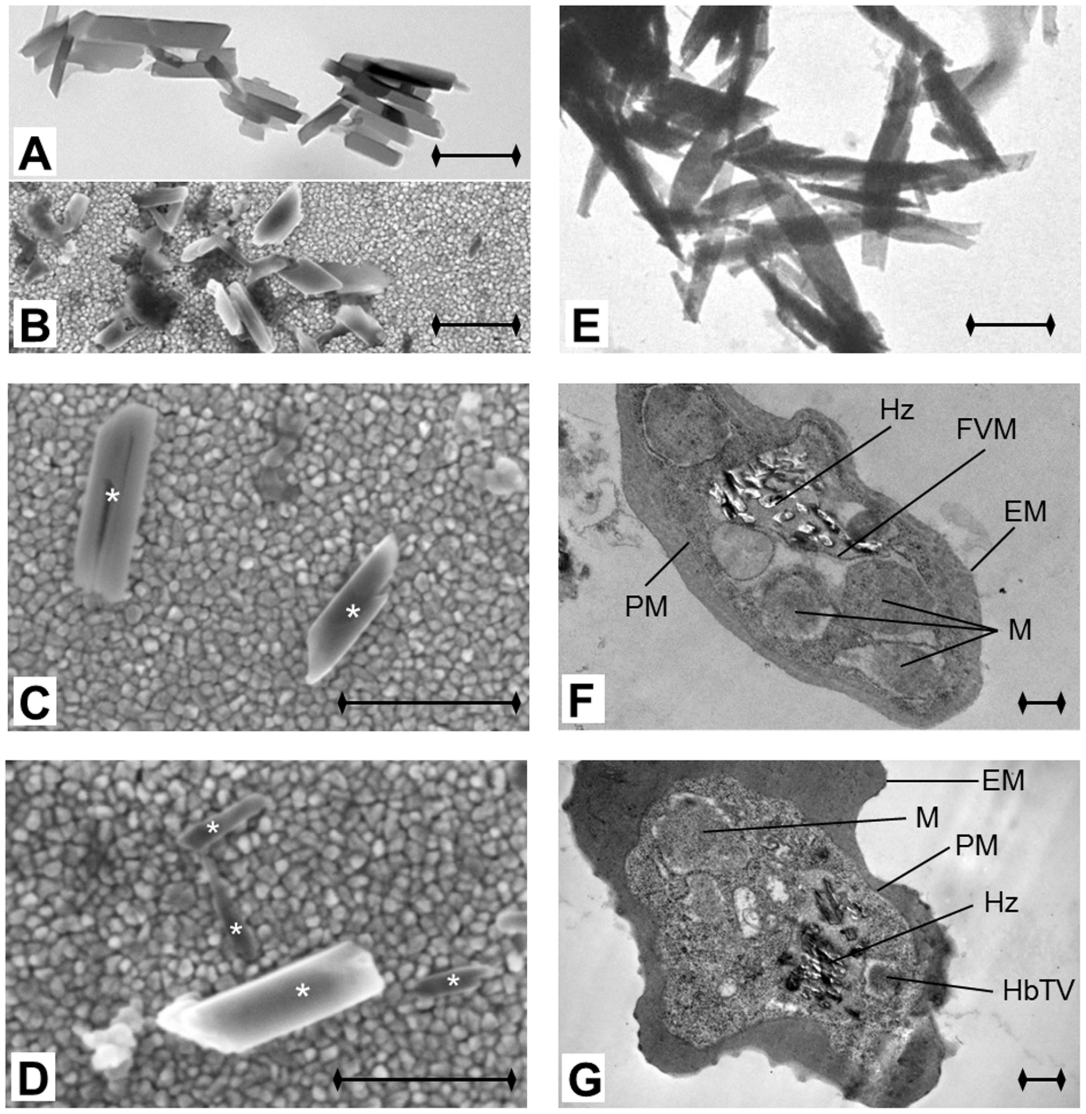
Hemozoin crystals observed by electron microscopy in-situ and after isolation. The transmission electron microscopy (TEM) image in panel A and scanning electron microscopy (SEM) images in panels B, C and D show hemozoin crystals extracted from the samples previously used for the MO measurements. The granular background of the SEM images comes from the gold coating of the glass substrate. Individual crystals in panels C and D are marked with asterisks. The length of the black bar at the bottom right corner of each panel is 500 nm. The typical length of the extracted hemozoin crystals ranges from 200 nm to 500 nm. For comparison, a TEM image of synthetic hemozoin crystals used in our previous MO study [15] is shown in panel E. These synthetic crystals have more elongated shape with a typical length of 500–900 nm. Panels F and G display TEM images of intact infected erythrocytes in the schizont stage. Distinct components of the parasite and the erythrocyte can be observed including the erythrocyte membrane (EM), parasite membrane (PM), food vacuole membrane (FVM), merozoites (M), hemoglobin transport vesicles (HbTV), knobs (K) and hemozoin (Hz).

The estimate based on the MO signal gives lower values for the hemozoin content of both cultures than the estimate based on the hemoglobin conversion rates quoted above. The comparison between electron microscopy images of natural and synthetic hemozoin crystals, shown in Fig. 3, implies that this difference arises from the different size of the two types of crystals. The natural crystallites in this study were considerably smaller (with typical lengths of ∼200–500 nm) than the synthetic ones (∼500–900 nm) used in our former work [15]. The weaker frequency dependence of the MO signal observed in the present study indicates that natural crystals are able to follow the rotation of the magnetic field up to higher frequencies than the synthetic ones due to their reduced size. Furthermore, the comparison between the natural crystals within the parasites and those extracted from the cultures confirms no major change either in the size or in the morphology of the crystals due to our lysis-sonication protocol.

We have also studied the change in the hemozoin concentration during the maturation of parasites using additional cultures. Our preliminary results (displayed in Fig. S2–S4 of the Supporting Information S1) show the gradual increase of the hemozoin content during the maturation of the parasites and imply that hemozoin is present in ring stages in agreement with recent works [31]–[33].

## Conclusions

The potential of exploiting hemozoin as a magnetic biomarker for malaria diagnosis has stimulated extended research over the last few decades. Taking advantage of the paramagnetic nature of hemozoin, several approaches have been proposed to improve the sensitivity of existing methods e.g., by magnetic separation of malaria infected erythrocytes from whole blood prior to diagnosis [16], [34]–[36]. More recently, new techniques have emerged, which directly use hemozoin as target material for magnetic diagnosis. These techniques include detection of depolarized side-scatter in flow cytometry [37], electrochemical magneto immunoassays [38], magnetically enriched surface enhanced resonance Raman spectroscopy [39], [40], acousto-optic detection [31] and magneto-optical detection using polarized light [17], [18], [41]. Among them, to the best of our knowledge, our rotating crystal MO diagnostic technique is the first realized in a cost-effective and compact format with excellent sensitivity.

In the present study, the detection limit of our rotating-crystal MO diagnostic technique was found to be ∼40 parasites/*µ*L and ∼10 parasites/*µ*L for ring and schizont stage parasites, respectively. These limits of detection are below the threshold currently achievable with RDT (>100 parasites/*µ*L) and are within the same range as the limits of expert microscopy for malaria diagnosis (5–50 parasites/*µ*L) [1]. For the present set of blood samples, which were kept frozen and thawed before the measurement, the performance of the method was limited by a residual MO signal due to some part of the lysed cell suspension. This residual MO signal obscures the genuine MO signal of hemozoin at parasite densities lower than the limits quoted above. Preliminary results indicate that for measurements on freshly lysed blood samples, which is the condition relevant to instant diagnosis, the detection limit of our rotating crystal MO platform could be further improved.

Limitations of our diagnostic technique include i) the possibility of false positive detections due to the presence of hemozoin in the blood, e.g. contained within white blood cells [42], for extended periods of time after an infection has been cleared and ii) the possibility of false negative results in case an infection only contains very early ring stage parasites with little or no hemozoin. Furthermore, methods targeting only hemozoin as a marker for infection cannot distinguish between different malaria species. However, the specificity of our MO diagnostic scheme, owing to variations in the typical size and morphology of hemozoin crystals produced by different species, needs to be evaluated by a comparative study on various *Plasmodium species*. We emphasize that only studies on field isolates will be able to elucidate the impact of these possible confounding factors and the present study is the basis for such field-based trials.

It is currently believed that without active case detection of asymptomatic malaria infections, malaria eradication will be impossible or very difficult to achieve [6]. However, there are no diagnostic tools for rapidly screening hundreds of people per day, on-site and with high sensitivity [43]. In principle, the rotating-crystal MO diagnostic method has the potential to fulfill these requirements as it is cost-effective, rapid and highly sensitive. However, rigorous field based assessment of the method’s performance as well as further method development will have to be conducted in order to judge the value of this method.

Our preliminary results (see the Supporting Information S1) show that the present methodology may provide an efficient in-vitro laboratory tool to test the susceptibility of the parasites to novel or clinically relevant antimalarial drugs by monitoring the effect of drugs on the rate of hemozoin formation [44]. The scope of this technique may also cover the study or diagnosis of other human diseases, such as schistosomiasis, which are also caused by blood-feeding organisms producing hemozoin similarly to malaria parasites [45]–[48].

## Materials and Methods

### Parasite Culture

*P. falciparum* parasites (laboratory adapted strain 3D7) were cultured following the method of Trager and Jensen with modifications [49]. The culture medium was RPMI 1640 with L-glutamine (GIBCO cat # 31800) supplemented with 2 mg/mL NaHCO (Merck, cat # 106329), 25 mg/L gentamicin (Pfizer, cat # 61022027), 50mg/L hypoxanthine (Calbiochem, cat # 4010), 25 mM HEPES (SAFC, cat # 90909C) and 10% pooled O+ human serum (mixed blood groups, Australian Red Cross Blood Service). Cultures were maintained at 4% hematocrit with changes of culture medium every 48 hours and diluted with uninfected O+ red blood cells when the parasitemia exceeded 5%. Parasites were maintained in an atmosphere of 5% CO_2_ and 1% O_2_ in N_2_. Parasite cultures were kept in stage synchrony by applying the 5% Sorbitol method, first described by Lambros and Vanderberg [50]. Hemozoin liberated from late stage parasites by the synchronization process was removed by washing the cells in RPMI medium after the Sorbitol induced cell lysis and before re-establishment of the culture. Cultures A and B were harvested ∼0 hour and ∼40 hours after synchronization of the culture and prepared for the MO study according to the following protocol.

Parasite stages and densities were determined by counting the number of parasites in the following 6 stages: early ring (ER), late ring (LR), early trophozoites (ET), late trophozoites (LT), early schizonts (ES) and late schizonts (LS). For this determination 5000 red blood cells were counted on Giemsa stained thin blood films. Parasite density values were calculated from the parasitemia values based on the assumption that 1 *µ*L of blood contains 5×10^6^ red blood cells at 50% hematocrit [1]. For both cultures, 2-fold dilution series were prepared in duplicate and adjusted to a volume of 200 *µ*L at a hematocrit of 50%, using human erythrocytes (Red Cross blood bank, Royal Melbourne Hospital, VIC, Australia). Blood lysates were prepared from the dilutions by freeze-thawing twice and were stored at −80°C until they were shipped for MO measurement to Hungary. The sample set also contained uninfected reference samples.

### Blood Treatment Prior MO Diagnosis

After thawing the lysates they were diluted 20-fold in distilled water, to give a final volume of 4 mL. After adding 100 *µ*L of a red cell lysis buffer (2.5 V/V% Triton X-100 (Sigma-Aldrich, Budapest, Hungary) in 0.1 M NaOH (Sigma-Aldrich, Budapest, Hungary)) the sample was sonicated for 30 minutes to dissociate potential aggregates prior to measurement. MO signals were recorded on 1 mL volumes taken from the samples immediately after sonication. The possible effect of storage was assessed by re-measuring samples after 20 to 120 minutes kept at room temperature and after 24 hours kept at 4°C.

### Magneto-optical Measurements

MO measurements were performed blinded. Signals were recorded using 1 mL from each lysed sample. The prototype of the rotating magnet setup as well as the underlying physical principles of the detection method are described in our previous study [15]. In brief, the sample is inserted into an assembly of permanent magnets arranged in a ring. This creates a strong homogenous magnetic field (*B* = 1 T) at the center where the sample is inserted and allows the alignment of the crystals. When the magnetic ring is rotated, the co-aligned hemozoin crystals follow this rotation. Polarized light from a laser diode is transmitted through the sample in the direction perpendicular to the plane of the rotating magnetic field. The rotation of the co-aligned dichroic crystals gives rise to a periodic change in the transmitted intensity (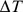), which – divided by the time-averaged intensity (*T*) – corresponds to the MO signal.

In fact, we demonstrated that the MO signal is proportional to the concentration of synthetic hemozoin crystals in the sample [15]. The MO signal was measured with increasing rotational speed values of the magnet, in the range of 1–130 Hertz. Our previous study showed that a good signal-to-noise ratio is observed in the range of 10–30 Hertz [15], and thus 20 Hertz was chosen to investigate the limit of detection. However, the measurement of the MO signal in the rotation frequency range of 1–130 Hertz was performed for two reasons: i) the frequency-dependence of the signal may provide information about the size distribution of the freely rotating crystals and ii) the frequency-dependence may give additional information at very low hemozoin concentrations and help to differentiate between infected samples and uninfected controls.

### Electron Microscopy

For transmission electron microscopy (TEM), parasite samples were fixed in resin blocks and 70–120 nm thin sections were cut using a Leica EM UC6 microtome (Leica Microsystems, North Ryde, NSW, Australia) and brought onto carbon coated copper TEM grids (ProSciTech, Thuringowa, Qld., Australia). The TEM grids were then stained with 5% uranyl acetate for 15 minutes and Reynold’s lead citrate solution for 5 minutes. For synthetic hemozoin, the aqueous suspension of crystals was dropped onto formvar membrane (purchased from Sigma-Aldrich) and brought onto carbon coated copper TEM grids. TEM was conducted on a JEOL 2100 TEM (JEOL Inc., Tokyo, Japan).

For scanning electron microscopy (SEM), the samples giving the highest MO signal were used and hemozoin crystals were extracted following the method of Chen and coworkers [45]. The dark brown pellet obtained by this method was re-suspended in 80 *µ*L water. For SEM imaging small droplets of the suspension containing the hemozoin crystals were applied to gold coated glass slides without further purification or treatment. The droplets were dried overnight at room temperature. The SEM images were acquired on a LEO 1540XB electron microscope using the in-lens detector. The accelerating voltage was set to 3 kV and the viewing angle was perpendicular to the gold surface. For details of the method see the Supporting Information S1.

## Supporting Information

Supporting Information S1 **Supporting information file containing Figs. S1–S4 and supporting text.**
(PDF)
