# Supplementary material for: Evaluation of a Novel Magneto-Optical Method for the Detection of Malaria Parasites
Source: PLoS One. 2014 May 13;9(5):e96981. doi: 10.1371/journal.pone.0096981 (PMC4019541; doi:10.1371/journal.pone.0096981)
Supplement: Supporting Information S1 — Supporting information file containing Figs. S1–S4 and supporting text. (PDF) [file pone.0096981.s001.pdf]

Supporting Information on

Evaluation of a novel magneto-optical method for the detection of malaria parasites

A. Orbán, A. Butykai, A. Molnár, Zs. Pröhle, G. Fülöp, T. Zelles, W. Forsyth, D. Hill, L. Schofield, I. Müller, M. Rebelo, T. Hänscheid, S. Karl, and I. Kézsmárki

The effect of sonication on the magnitude of the MO signal. To investigate the effect of freeze-thawing and sonication we performed a preliminary experiment comparing the MO signal magnitude measured on three samples obtained from the same parasite culture but treated in three different ways before the MO measurement. An unsynchronized *P. falicparum* (3D7) culture with a parasitemia of 1 % was used, cultured as described in the Materials and Methods section. The culture were centrifuged and after removing the supernatant they were diluted with PBS to 50 % hematocrit and divided into three parts which were prepared differently:

(1) One sample was frozen and thawed twice, subsequently frozen again and after the third thawing diluted 20-fold with distilled water, treated with the clearing solution and sonicated for 30 minutes. *Thus, this sample was treated the same way as the samples of the dilution series described in the article.*

(2) The second sample was only diluted with distilled water and treated with the clearing solution for 30 minutes, without sonication.

(3) The third sample was diluted with distilled water and treated with the clearing solution for 30 minutes while being sonicated.

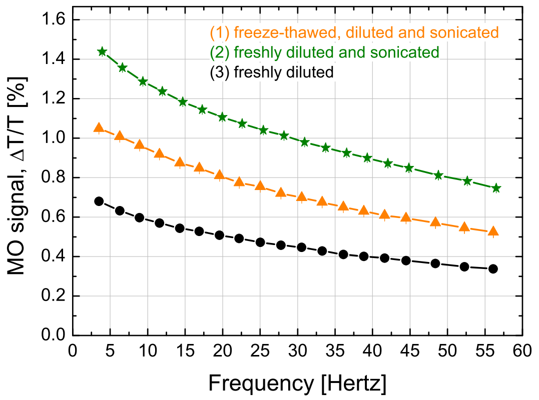

FIG. S1: The effect of sample preparation on the magnitude of the MO signal. The frequency dependent MO signals for triplicate samples from the same *P. falciparum* culture were measured after preparations (1)-(3) as described in the text.

The results in Fig. S1 show that sonication increases the MO signal, while the signal is reduced by the freeze-thaw process.

Detection of increasing hemozoin concentrations during maturation of synchronized ring-stage *P. falciparum* cultures. *P. falciparum* cultures (3D7) were maintained as described in the Materials and Methods section with modifications concerning the synchronization process. In addition to the sorbitol synchronization, the cultures were exposed to a gelatine sedimentation/floatation process[1] when the majority of the parasites were in the schizont stage. This technique allows the enrichment of knob-infected erythrocytes, which correspond to young schizonts. After incubating the parasites obtained with this method under standard conditions for 24 hours two cultures (culture C and D) were synchronized using sorbitol. After the synchronization a volume of 1.2 mL from both cultures was used for the first MO measurements, and the cultures were re-incubated. These MO samples are referred to as the 0 hour samples. Before the MO measurement Giemsa stained thin blood films were prepared from the samples. The parasite stage distribution was determined by counting 5000 erythrocytes, some of which were also observed using conventional and polarization microscopy to check for the presence of hemozoin. After 3, 4 and 6 hours of incubation other 1.2 mL samples were taken from each culture for further MO measurements, before which additional Giemsa stained thin blood films were prepared from the samples to confirm parasite maturation. All samples for MO measurements were centrifuged for 5 minutes at 1800 rpm and the pelleted red blood cells were re-suspended in PBS to 50 % hematocrit. These blood samples were diluted 10 times with distilled water and treated with the clearing solution for 30 minutes prior to the MO measurements.

As shown in Fig. S2, cultures C and D consisted of early-ring (ER), late-ring (LR) and early-trophozoite (ET) forms and had a parasitemia of 2.8 % and 1.5 %, respectively. Culture C contained mainly rings, especially late rings, but only around 10 % early trophozites. The stage distribution in culture D showed more early trophozoites and less late rings than in culture C. Fig. S3 shows the frequency dependent MO signal for cultures C and D measured 0, 3, 4 and 6 hours after synchronization. As expected, no parasite replication occurred in this pe-

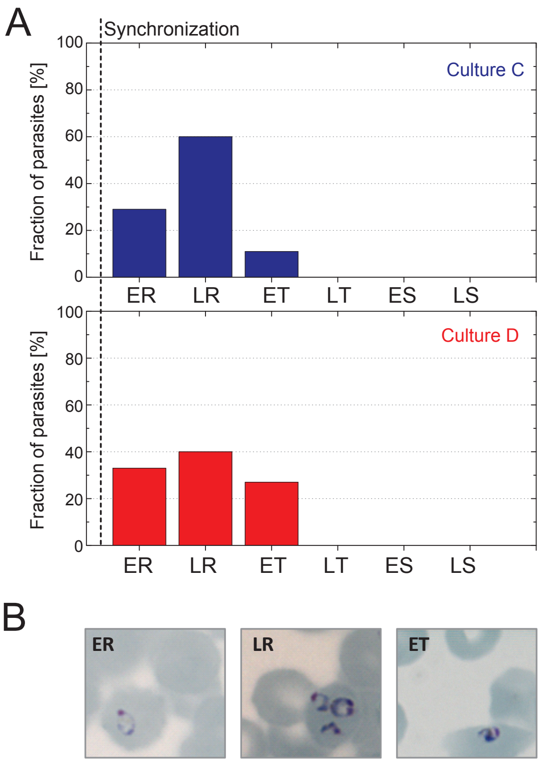

FIG. S2: Panel A: Distribution of parasite developmental stages in the *P. falciparum* cultures C and D. Panel B: Light microscopy images of Giemsa stained thin blood films containing infected red blood cells with parasites in different stages of maturity (taken from these two cultures). In both panels the labels ER, LR, ET correspond to early-ring, late-ring, early-trophozoite stages, respectively.

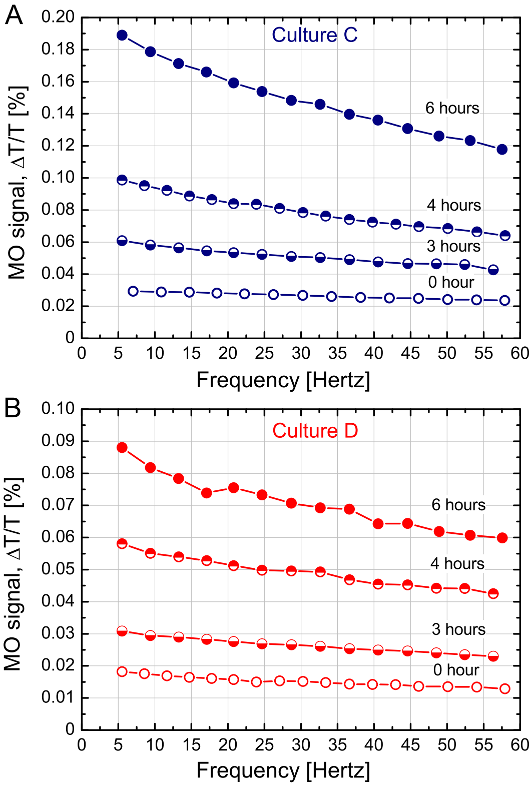

FIG. S3: Magneto-optical detection of the hemozoin production during the maturation of cultures C and D displayed in panel A and B, respectively. Measurement of the frequency dependent MO signal was performed 0, 3, 4, and 6 hours after the synchronization.

riod, as was confirmed by counting identical parasitemias on thin blood smears at each time point. The increase in the signal of the MO measurements at 3, 4 and 6 hours in both cultures indicates that the signal is caused by a dynamic process and not by measuring some static remnant in the culture. Thus, the increase in the MO signal is due to the production and consequently increasing amounts of hemozoin produced by these early stages. It means that the method is able to follow parasite maturation in clinically relevant time-frame, that can eventually be applied to develop possible fast in vitro drug susceptibility assays.

The time evolution of the MO signal (at the rotation frequency of 20 Hertz) for the two cultures is compared in Fig. S4. We found that the MO signal increases with the same rate for the two cultures, although culture D contains 27 % early trophozoites and culture C only 10 %. This indicates that in both cultures ring stages have a major contribution to the hemozoin production in the time window (0-6 hours) investigated here. Interestingly, hemozoin crystals were not visible when the thin blood smears were observed with either conventional or polarization microscopy at 0 hour, indicating that crystals may be too small to be observed by these methods.

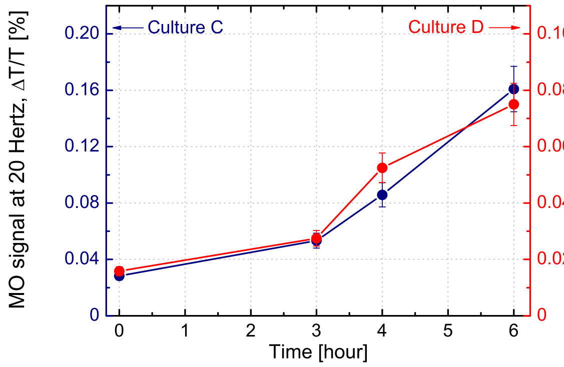

FIG. S4: Magneto-optical detection of the hemozoin production during growth of ring-form synchronized *P. falciparum* cultures C and D. The MO signal measured at 20 Hertz is plotted for the time points 0, 3, 4 and 6 hours. Note that the vertical scales are different for cultures C and D with different parasitemia levels. The error bars indicate the maximal deviations between duplicate samples.

Sample preparation for electron microscopy. For transmission electron microscopy (TEM), para-

site samples were fixed in 2 % glutaraldehyde, 1 % paraformaldehyde in PBS for 60 min at 4^oC and washed twice with PBS (pH=7.4) in 1.5 mL Eppendorf tubes. Subsequently, cells were suspended in 1 % Osmium tetroxide for 1 h. Samples were then dehydrated using a PELCO Biowave microwave processor (TedPella Inc., Redding, CA, USA) by passage through increasing ethanol concentrations in water (33 %, 50 %, 66 %, 100 %) followed by two washes in dry acetone. Subsequent to dehydration, samples were resin infiltrated again using the PELCO Biowave microwave processor and passage through increasing resin concentrations in acetone (33 %, 50 %, 66 %) followed by two incubations with pure resin. The resin used was a mixture of araldite and dodecenyl succinic anhydride (DDSA) to which a polymerizer (benzyldimethylamine, BDMA) was added. The resin infiltrated samples were cured at 70^oC overnight in a laboratory oven (Thermo Fisher Scientific, Scoresby, Victoria, Australia). The cured resin blocks were then removed from the Eppendorf tubes and 70-120 nm thin sections were cut using a Leica EM UC6 microtome (Leica Microsystems, North Ryde, NSW, Australia) and brought onto carbon coated copper TEM grids (ProSciTech, Thuringowa, Qld., Australia). The TEM grids were then stained with 5 % uranyl acetate for 15 min and Reynold's lead citrate solution for 5 min. TEM was conducted on a JEOL 2100 TEM (JEOL Inc., Tokyo, Japan).

For scanning electron microscopy (SEM), the samples giving the highest MO signal were used and hemozoin crystals were extracted following the method of Chen and coworkers.[2] The RBC lysis protocol was slightly modified, since the original blood samples were already diluted and treated with the clearing solution prior to the MO measurements as described above. In the first step of the extraction process the total sample volume of 4 mL was sonicated, vortexed and then equally divided into four Eppendorf tubes. The samples were ultracentrifuged (12,000 rpm for 15 min) and the transparent supernatant was removed. The remaining dense fraction was sonicated in 2% SDS and from this point the protocol of Chen and coworkers was followed.[2] After the incubation in 6 M of urea the sample was washed three times in SDS and three times in distilled water. In the last washing step the supernatant was removed and the clearly visible dark brown pellet was resuspended in 80 μL water. For SEM imaging small droplets of the suspension containing the hemozoin crystals were applied to gold coated glass slides without further purification or treatment. The droplets were dried overnight at room temperature. The SEM images were acquired on a LEO 1540XB electron microscope using the in-lens detector. The accelerating voltage was set to 3 kV and the viewing angle was perpendicular to the gold surface.

[1] Goodyer ID, Johnson J, Eisenthal R, Hayes DJ. Purification of mature-stage Plasmodium falciparum by gelatine flotation. (1994) Ann Trop Med Parasitol 88(2): 209-211.

[2] Chen MM, Shi L, Sullivan DJJr, et al. Haemoproteus and Schistosoma synthesize heme polymers similar to Plasmodium hemozoin and beta-hematin. (2001) Mol Biochem Parasitol. 113(1):1-8.
